# MCT4 is an important determinant for the growth of highly glycolytic and aggressive malignancies

**DOI:** 10.1186/1753-6561-6-S3-O26

**Published:** 2012-06-01

**Authors:** Kezi Unsal-Kacmaz, Shoba Ragunathan, Jennifer Shan, Bing Guo, Xiaoli Wang, Diane Tkach, Judy Lucas, Anke Klippel

**Affiliations:** 1 Oncology Research Unit, Pfizer Oncology, Pfizer Worldwide Research and Development, 401 N. Middletown Road, Pearl River, NY 10965, USA

## 

Constitutive upregulation of glycolysis is likely to be an adaptation to hypoxia that develops as premalignant lesions grow progressively distant from their blood supply. Increased lactic acid production caused by the upregulation of glycolysis results in microenvironmental acidosis. Cell populations that emerge from this adaptation have a powerful growth advantage, as they alter their environment through increased glycolysis in a way that is toxic to other phenotypes, but harmless to themselves or their re-programmed stromal environment. Our goal is to elucidate the molecular basis for this aberrant behavior, and harness that knowledge towards the development of effective therapeutic strategies against malignant tumors. Here we describe in vitro/in vivo functional analysis of MCT1, MCT4 and CD147 in three different cell contexts, where MCT1 and MCT4 are expressed either alone or together and confer differential growth advantages under hypoxic or normoxic conditions. We show the selective enrichment of CD147 and MCT4 in highly tumorigenic CD133/CD44 positive cells from colon cancer patient-derived-xenografts.

Our findings indicate that MCT4 is an important determinant for the growth of highly glycolytic and aggressive malignancies, which so far has not been explored as a target for therapeutic purposes and may provide a unique avenue for anticancer therapy. We will provide *in-vitro* and *in-vivo* evidence for tumor contexts, where the selective inhibition of MCT4 is sufficient as well as contexts, for which the combined inhibition of MCT1 and MCT4 is required for providing a therapeutic benefit.

